# Lath formation mechanisms and twinning as lath martensite substructures in an ultra low-carbon iron alloy

**DOI:** 10.1038/s41598-018-32679-6

**Published:** 2018-09-24

**Authors:** D. H. Ping, S. Q. Guo, M. Imura, X. Liu, T. Ohmura, M. Ohnuma, X. Lu, T. Abe, H. Onodera

**Affiliations:** 1 National Institute for Materials Science, Sengen 1-2-1, Tsukuba, 305-0047 Japan; 2 Faculty of Engineering, Hokkaido University, Kita 13 Nishi8, Kita-ku, Sapporo, Hokkaido 060-8628 Japan; 3 School of Materials Science and Engineering, Dalian Jiaotong University, Dalian, 116028 China

## Abstract

Lath martensite is the dominant microstructural feature in quenched low-carbon Fe-C alloys. Its formation mechanism is not clear, despite extensive research. The microstructure of an Fe-0.05 C (wt.%) alloy water-quenched at various austenitizing temperatures has been investigated using transmission electron microscopy and a novel lath formation mechanism has been proposed. Body-centered cubic {112}〈111〉-type twin can be retained inside laths in the samples quenched at temperatures from 1050 °C to 1200 °C. The formation mechanism of laths with a twin substructure has been explained based on the twin structure as an initial product of martensitic transformation. A detailed detwinning mechanism in the auto-tempering process has also been discussed, because auto-tempering is inevitable during the quenching of low-carbon Fe-C alloys. The driving force for the detwinning is the instability of ω-Fe(C) particles, which are located only at the twinning boundary region. The twin boundary can move through the ω ↔ bcc transition in which the ω phase region represents the twin boundary.

## Introduction

Martensite transformation mechanisms have long been discussed owing to their important role in understanding the various microstructures formed in steels, particularly in the quenched state of Fe-C alloys with different levels of carbon. A large, plate-like form of quenched martensite with a body-centered cubic (BCC) {112}〈111〉-type twin structure as its substructure is easily formed in high-carbon steels or alloys with a low M_s_ (martensite start temperature), whereas the martensite in low-carbon steels, normally called lath martensite, has been suggested to have a dislocation structure as its lath substructure after quenching. These different substructures have resulted in the suggestion of various kinds of transition mechanisms from austenite (γ-Fe) to ferrite (α-Fe)^1–10^.

Recently, the possibility of a twin structure being a lath martensite substructure has been experimentally confirmed in an oil-quenched low-carbon Fe-0.1C (wt.%) alloy^11^. The twin structure is also common in other quenched low-carbon martensites such as Fe-Ni-C^12–14^; however, the twin formation mechanism (BCC {112}〈111〉-type twin formed in austenite with face-centered cubic (FCC) structure) is still unclear, although the related research activity started in the early 20th century^15–17^. In particular, the mechanism concerning the twins remaining inside the laths after quenching has not been well explained.

In order to confirm whether a twin can be observed in ultra-low-carbon alloys, the microstructure of an Fe-0.05 C (wt.%) alloy subjected to water-quenching at temperatures from 1050 °C to 1350 °C has been examined using transmission electron microscopy (TEM). The C content (0.05 C) in the Fe-C binary alloy is close to that of industrial pure iron (0.02C).

Although a twin structure was observed as the lath martensitic substructure in quenched Fe-0.1C (wt.%) samples, a twin was not observed in quenched Fe-0.05 C (wt.%) samples owing to the low austenitizing temperature of 950 °C employed in our previous work^11^. Thus, in the present investigation, the austenitizing temperature has been increased to explore the twin structure in quenched Fe-0.05 C (wt.%) alloys. A new lath formation mechanism has been discussed at atomic scale for easy understanding.

## Results

Fe-0.05 C samples subjected to water-quenching after heat treatment at 950 °C were investigated^11^. Typical laths with fine cementite particles at the lath boundaries were observed in the quenched state, and no trace of twinning structure could be observed.

### Austenitizing temperature ~1200 °C

**Figure 1:**
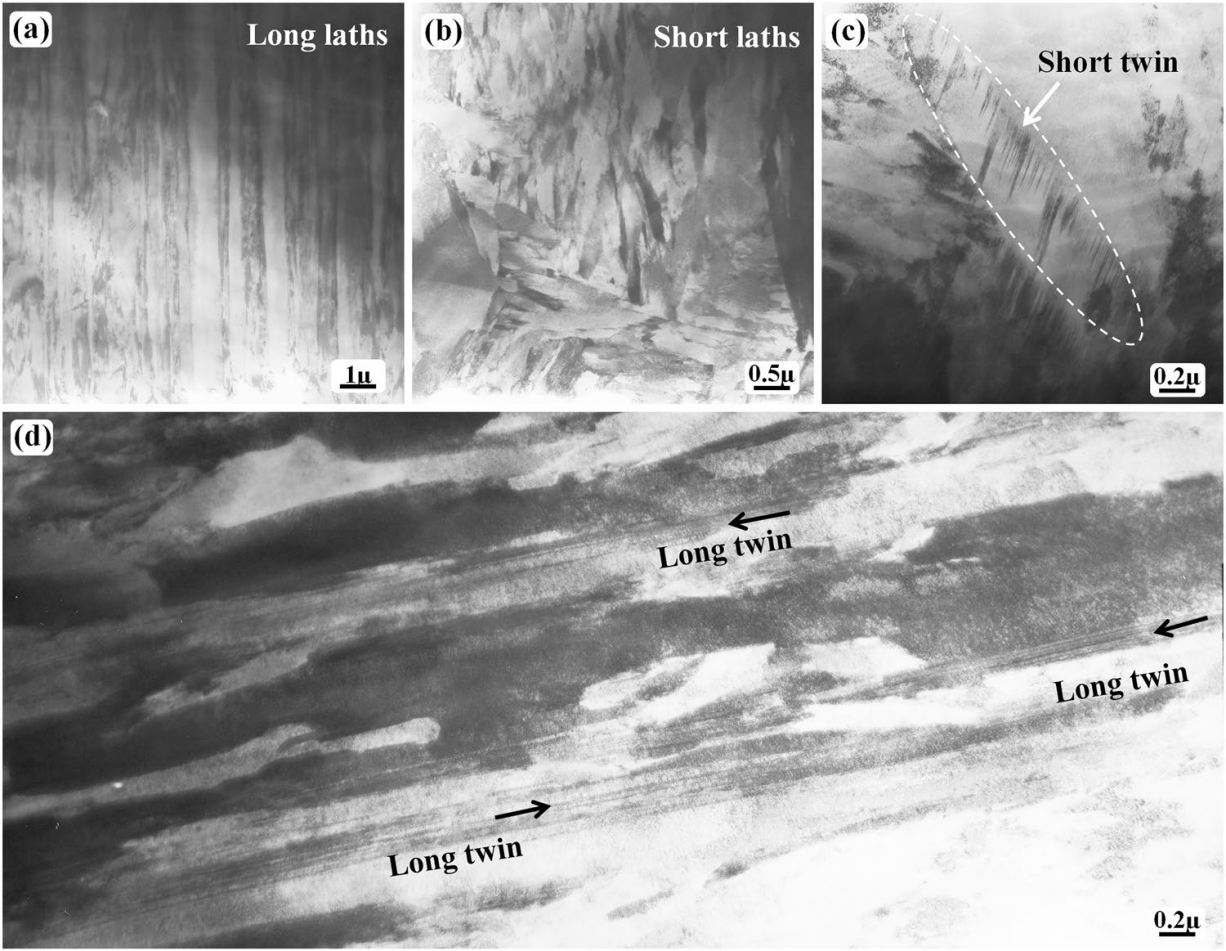
Bright-field TEM images of the water-quenched Fe-0.05 C sample (~500 µm in thickness) after austenitizing at 1200 °C for 30 h. (**a**) General lath-type morphology. (**b**) Short lath morphology. (**c**) Twin contrast inside the laths. (**d**) Long twin morphology.

Figure 1 shows the general microstructure in the quenched state of the sample austenitized at 1200 °C for 30 min. All the images (Fig. 1(a–d)) are obtained from the same TEM specimen. Typical lath-type martensite morphology can be observed in Fig. 1(a); the lath width is approximately several hundreds of nanometers and its length is tens of microns or more. A lath-type microstructure is frequently observed; the most common microstructure, shown in Fig. 1(b), has a shorter and wider lath morphology compared with that shown in Fig. 1(a).

A regular array is evident for the laths shown in Fig. 1(a), whereas the short laths show more or less disordered arrangements. A twin contrast is frequently observed inside the short laths (Fig. 1(c)). This kind of twin can be referred to as the lath martensitic substructure and is generally called the “internal” twin^12–14,18–20^. This type of twin is sometimes referred to as the “short internal twin”. This is because another type of twin structure morphology can be observed in Fig. 1(d), whose length is long, similar to the typical lath length; the length direction of this kind of twin, called the “long twin”, is parallel to that of typical laths. The short twin (internal twin) is inside a lath, whereas the long twin is independent of any lath; the long twin itself can be treated as a long lath. The twin contrast is absent in some martensite, but this does not imply that there are no twins. This is because the twinning boundary is only one of the {112} planes (not every 112 plane can be the twinning plane) in a BCC-structured martensite. No twin diffraction contrast can be observed if the twinning plane is inclined significantly to the incident electron beam^21,22^. Notably, the twinning plane or boundary of the short twin is normally inclined to the lath boundary, in contrast to the long twin whose twinning plane is parallel to the lath boundary.

**Figure 2:**
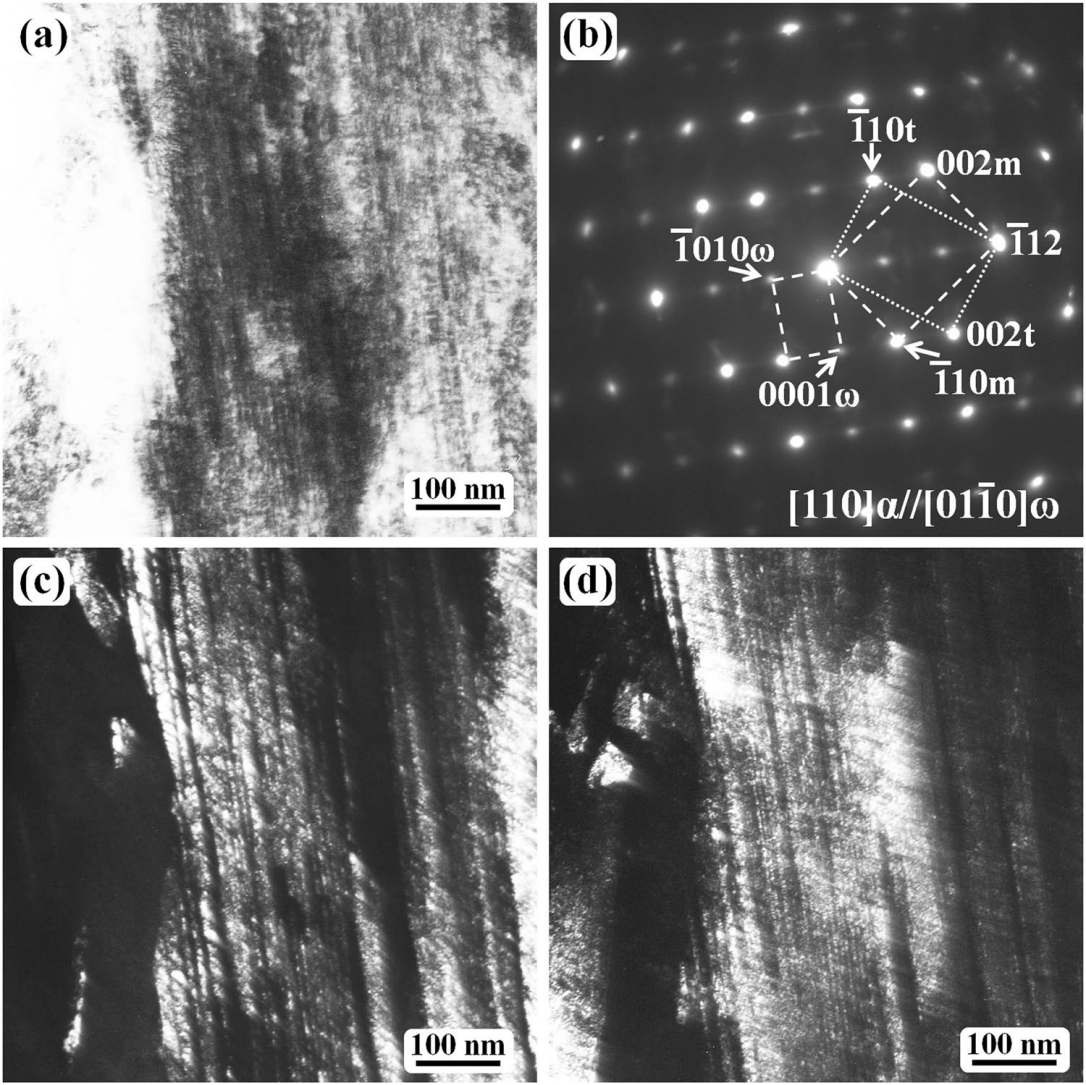
TEM results of the water-quenched Fe-0.05 C samples after austenitizing at 1200 °C for 30 min. (**a**) Bright-field micrograph revealing the long twin contrast. (**b**) SAED pattern showing the twinning diffraction pattern with the electron beam parallel to the [110] α-Fe direction. (**c**) The dark-field image obtained using the $\bar{1}$10_m_ matrix spot. (**d**) The dark-field image of the $\bar{1}$10_t_ twin spot.

The twinning crystal structure is the same in both the short internal twin and long twin. Figure 2 shows the structure of the long twin. When the [110]_α_ zone axis of the twinning structure is parallel to the incident electron beam, the diffraction contrast in the bright-field mode (Fig. 2(a)) is not sufficient to distinguish the twin morphology owing to the high density of thin twins. Thus, the dark-field images showing the matrix crystal and twin crystal are shown in Fig. 2(c,d), respectively.

The selected-area electron diffraction (SAED) pattern (Fig. 2(b)) shows a typical BCC {112}〈111〉-type twin structure with extra spots at 1/3($\bar{1}$12) and 2/3($\bar{1}$12), which are the diffraction spots from a metastable hexagonal ω-Fe phase^21–27^. Both the matrix crystal and twin crystal are thin with a thickness of several nanometers or tens of nanometers, and the twin boundaries seem to be straight or flat. However, the twinning boundaries are curved for the short internal twin (Fig. 1(c)), and the curved boundaries are very likely formed on the detwinning path, and close to the end of the detwinning process. During the detwinning process, different regions of the twin boundaries may have a different moving speed; thus, the boundaries are curved. The difference at different twin boundary region is dependent on the number and density of the omega particles.

**Figure 3:**
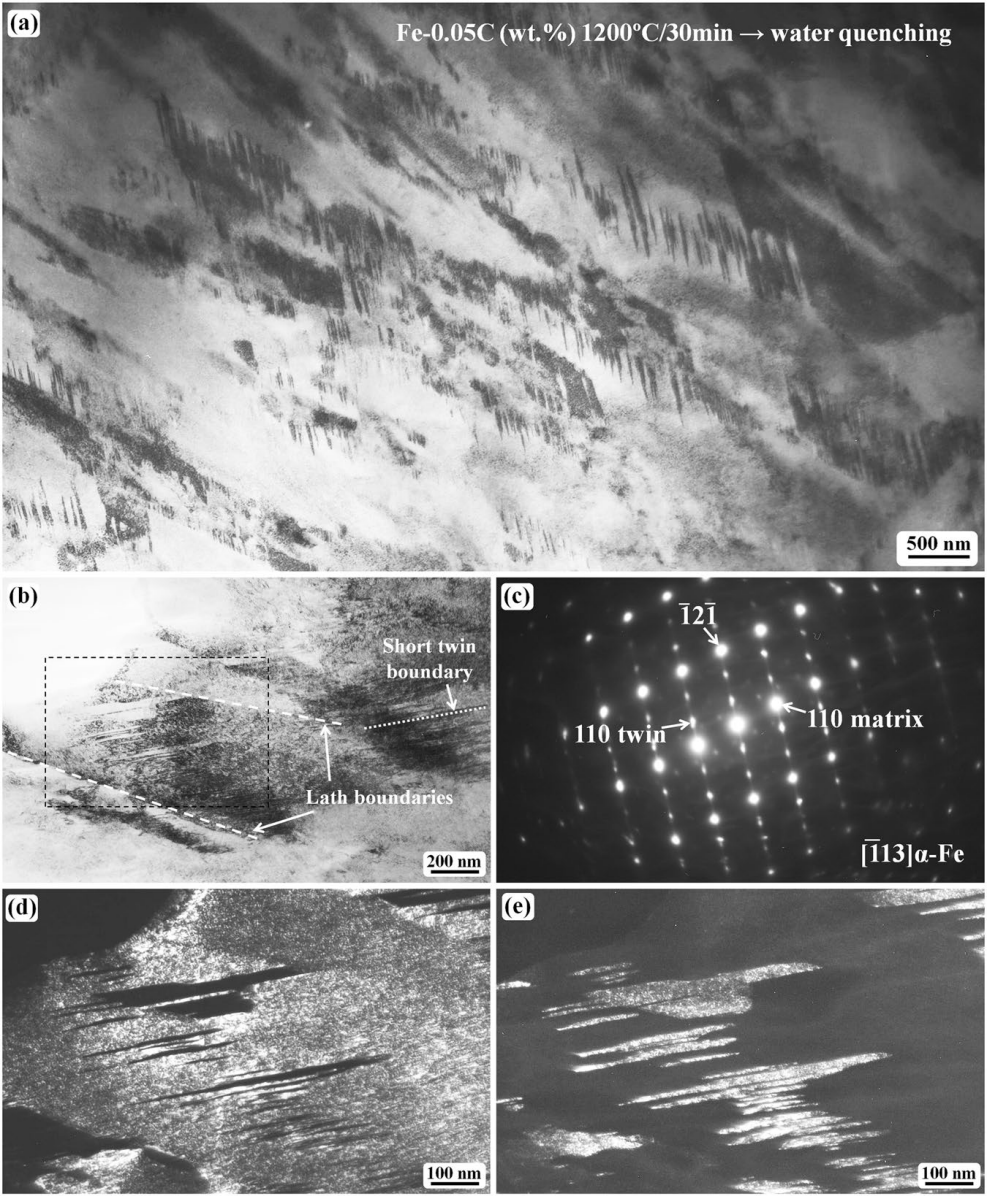
Short twin boundary morphology of the water-quenched Fe-0.05 C samples after austenitizing at 1200 °C for 30 min. (**a**) Bright-field micrograph revealing the short twin contrast inside the laths. (**b**) Bright-field image of a local area with the [$\bar{1}$13] α-Fe zone axis parallel to the incident electron beam. (**c**) The corresponding SAED pattern of (**b**). (**d**) The dark-field image obtained using the 110 matrix spot. (**e**) The dark-field image of the 110 twin spot. (**d**,**e**) are obtained from the region outlined by the dashed dark rectangle line in (**b**).

Figure 3 shows the TEM results illustrating the morphology of the short twin boundary. Figure 3(a) shows a general bright-field image revealing that short twins commonly exist inside laths. Each lath has a short twin structure as its substructure; the twin contrast may be absent in some laths owing to the mis-orientation of the laths. As the twinning plane is one of the BCC {112} planes, the twin contrast is expected to be clearly observed from only the twinning plane which is parallel to the incident electron beam. A tilting experiment was carried out to confirm that the twin contrast can be observed in each lath in the present sample, and that the twinning plane or boundary of the short twin is normally inclined to the lath boundary. Figure 3(b) shows a partial lath region with the [$\bar{1}$13]_α_ zone axis (Fig. 3(c)) parallel to the incident electron beam. The SAED pattern (Fig. 3(c)) and the dark-field images (Fig. 3(d,e)) confirm that the twinning plane is parallel to the incident electron beam; otherwise, the twin boundary would not show a clear contrast. The dark-field image obtained from the matrix 110 diffraction spot (Fig. 3(d)) shows that short twins are embedded inside the lath (the twins are the dark plates inside the bright lath). The dark regions inside the lath correspond to the twin crystals. The dark-field image obtained from the twin spot (Fig. 3(e)) shows that the twins (the bright regions inside the lath) are curved, without straight or flat twinning boundary planes. The morphology of the short twin boundary is different from that of the long twin, which has more straight or flat boundary planes.

**Figure 4:**
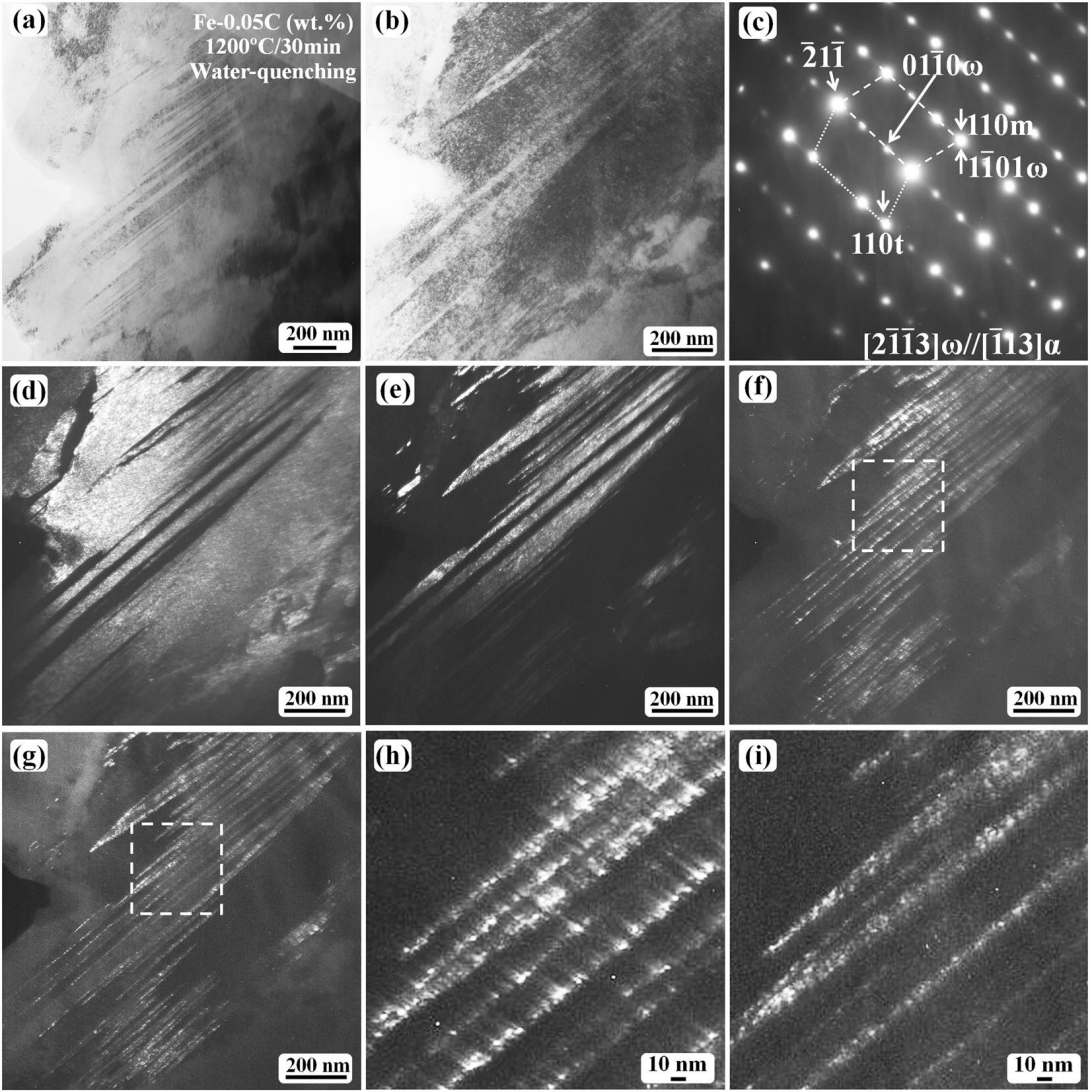
Short twin boundary morphology of the water-quenched samples after austenitizing at 1200 °C for 30 min. (**a**) Bright-field micrograph revealing the short twin contrast inside laths. (**b**) The region (**a**) after being tilted toward the (**c**) zone axis condition. (**c**) The SAED pattern showing twinning diffraction pattern along [$\bar{1}$13] α-Fe direction. (**d**) The dark-field image obtained using the 110 matrix spot. (**e**) The dark-field image of the 110 twin spot. (**f**,**g**) are the dark-field images corresponding to the spots 02 $\bar{2}$0 ω and 01 $\bar{1}$0 ω in (**c**), respectively. (**h**,**i**) are the enlarged images of the regions outlined in (**f**,**g**), respectively.

In order to observe the size and morphology of the ω-Fe phase, a thin region with the short twins has been analyzed as shown in Fig. 4. Figure 4(a) shows a TEM bright-field image revealing the short internal twins inside the laths. The twin contrast can be observed when the diffraction is off the [$\bar{1}$13]_α_ zone axis; however, the twin boundary is inclined to the incident electron beam direction at this condition. Once the twinned region is tilted toward the [$\bar{1}$13]_α_ zone axis, it becomes difficult to observe the twinning boundary contrast owing to the dark contrast of both the matrix and the twin, as shown in the bright-field image (Fig. 4(b)); the corresponding SAED pattern is shown in Fig. 4(c). Thus, the dark-field observation is necessary to view the twin contrast. The needle-like morphology of the short internal twin can be recognized from the dark-field images (Fig. 4(d,e)). The ω-phase particles are distributed at the twinning boundary regions as revealed in Fig. 4(f,g). The ultra-fine particle size of the ω-phase is estimated to be 1–2 nm from the enlarged images (Fig. 4(h,i)). As the ω-particles are at the twinning boundary region, we can recognize the morphology of the short internal twin from the ω-particle distribution. The short internal twin is needle-like with a curved surface, which is different from the morphology of the long twin.

### Austenitizing temperature ~1250 °C

**Figure 5:**
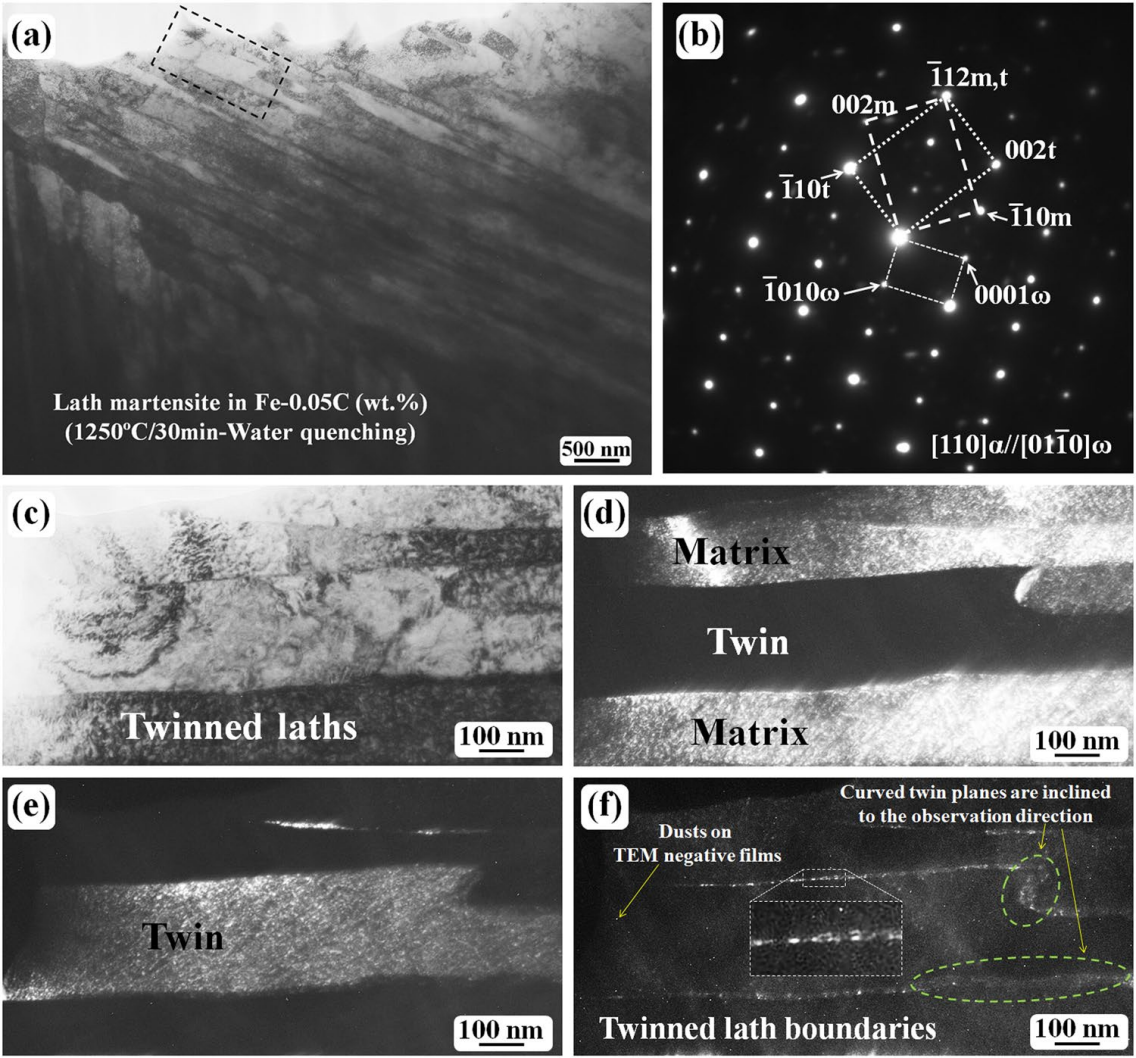
Twinned laths of the water-quenched Fe-0.05 C samples after austenitizing at 1250 °C for 30 min. (**a**) Bright-field micrograph showing the lath morphology. (**b**) The SAED pattern revealing that the laths in (**a**) have a BCC {112}〈111〉-type twinning relationship. (**c**) An enlarged image of the region outlined by the black dashed line in (**a**). (**d**) The dark-field image obtained using the $\bar{1}$10 matrix spot. (**e**) The dark-field image of the $\bar{1}$10 twin spot. (**f**) The dark-field image obtained using the 0110 ω-Fe spot. A local lath boundary contrast is enlarged and shown in the dashed line region in (**f**).

The effect of a higher austenitizing temperature on the twin structure and martensite morphology of the water-quenched Fe-0.05 C (wt.%) alloys has also been investigated. Figure 5 shows the general microstructure in the quenched state at 1250 °C after the sample was treated for 30 min. No trace of the twin substructure can be observed inside the laths. However, a twinned lath was observed and an example is shown in Fig. 5.

Figure 5(a,b) show the lath morphology and the corresponding SAED pattern ([110]_α_ zone axis), respectively. The diffraction pattern (Fig. 5(b)) reveals a BCC {112}〈111〉-type twinning structure among the laths. Figure 5(c) shows a high-magnification image of the local region outlined by a black dashed line in (a). The dark-field images obtained using the $\bar{1}$10 m spot and $\bar{1}$10 t spot in (b) are shown in Fig. 5(d,e), respectively. Thus, the region with bright contrast corresponds to the matrix crystal, whereas the region with dark-contrast in Fig. 5(d) corresponds to the twin crystal. In Fig. 5(e), the twin has a bright contrast. Figure 5(f) shows a dark-field image obtained from the 0110_ω_ diffraction spot. The inset in Fig. 5(f) is a high-magnification image of the local region outlined by dashed lines. This enlarged image reveals that the ω-Fe phase has a fine particle-like morphology, and also that the ω-Fe fine particles are distributed at the twinned lath boundary region^27^. After the picture is enlarged, fine dusts on the TEM negative films become evident as indicated by the arrow. As the twinned lath boundaries are curved, the boundary plane is not parallel to the observation direction; thus, the boundary plane becomes wide in some regions.

### Austenitizing temperature ≥1300 °C

**Figure 6:**
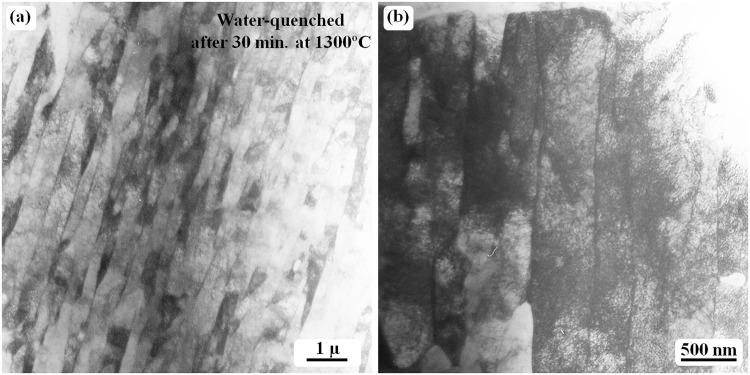
TEM bright-field images obtained from the Fe-0.05 C sample water-quenched at 1300 °C. (**a**) Lath martensite structure, which is free from any carbide or twin. (**b**) The absence of any carbide at the lath boundaries after the specimen was tilted by approximately 10°. The titling experiment was used to confirm the existence of carbide particles at the boundaries.

The microstructure in the sample quenched at 1300 °C is similar to that observed in the sample quenched at 1250 °C. The general images are shown in Fig. 6(a). Laths with twinning relationships are commonly observed, as shown in Fig. 6(b), which is similar to the image shown in Fig. 5(a). No obvious carbides can be observed.

**Figure 7:**
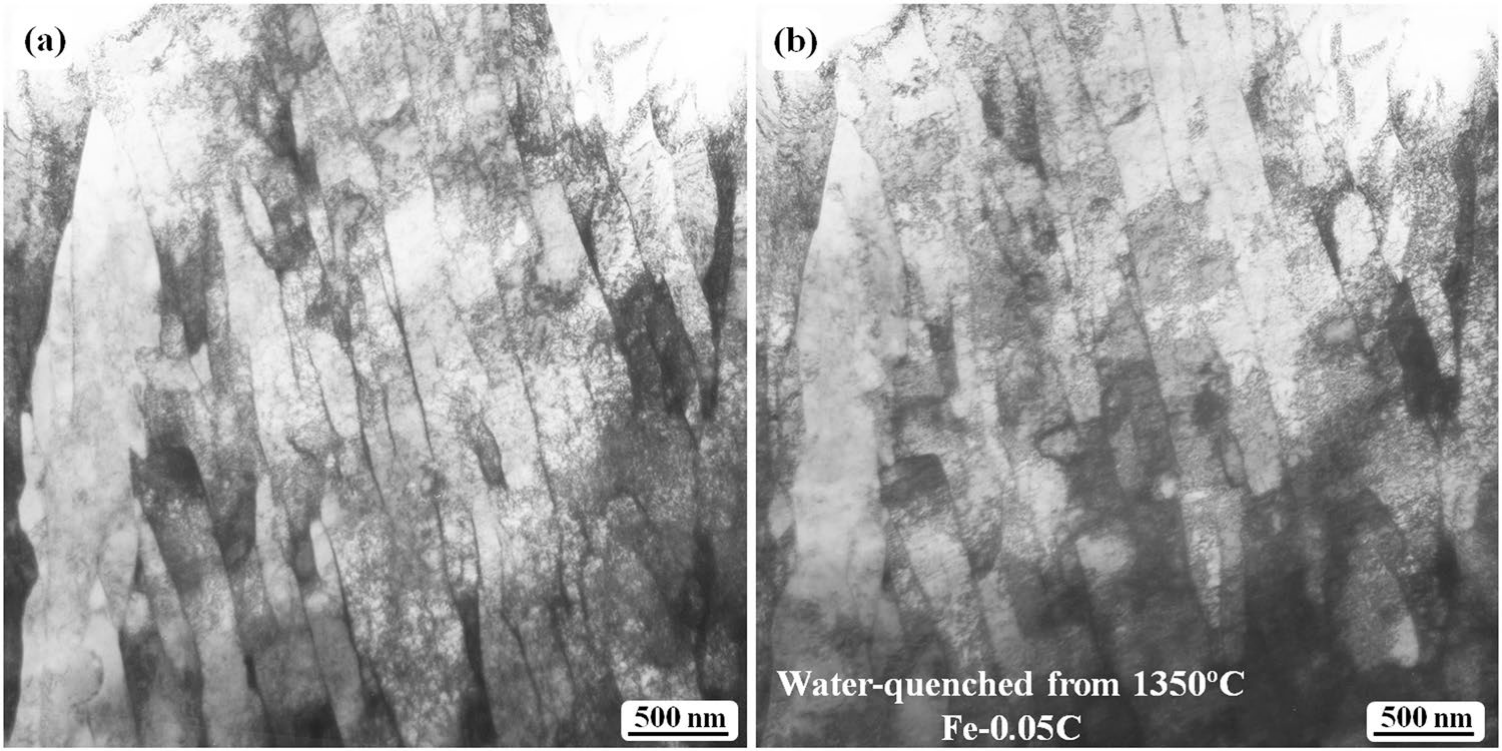
TEM bright-field images obtained from the Fe-0.05 C sample water-quenched at 1350 °C. (**a**) Lath martensite structure free from any carbide or twin. (**b**) Image showing the absence of any carbide at the lath boundaries after the specimen was tilted by approximately 10°. Both images are obtained from the same region.

The absence of twin structures inside the laths is most likely due to decarbonization, which may result in lower carbon content in the samples at a temperature higher than 1200 °C during the present treatment (in flowing Ar in a furnace at the high temperature). TEM analysis was also performed on the samples quenched at 1350 °C; no trace of twins (twinned laths or twins as lath substructures) can be observed in these samples (Fig. 7(a)). TEM tilting was carried out to observe whether there were fine cementite particles at the lath boundaries (Fig. 7(b)). Figure 7(a) shows a typical region with several lath boundaries; the same region is shown in Fig. 7(b) after being tilted by approximately 10°. Both images show that fine particle contrast cannot be observed anywhere, including at the lath boundaries, which indirectly suggests that the carbon content is much lower than 0.05 (wt.%). This conclusion has been reached simply by comparing the present results with those obtained for the samples quenched at 950 °C, which revealed a high density of fine cementite particles at the lath boundaries^11^.

To confirm the decarbonization at high temperature, an *in-situ* heating TEM observation was also carried out on a water-quenched sample at 1350 °C. No obvious carbide particles could be observed via TEM after the quenched sample was *in-situ* heated to 600 °C. This experimental result suggests that less carbon remained in those samples water-quenched at the high temperature (≥1300 °C).

### Austenitizing temperature ~1050 °C

**Figure 8:**
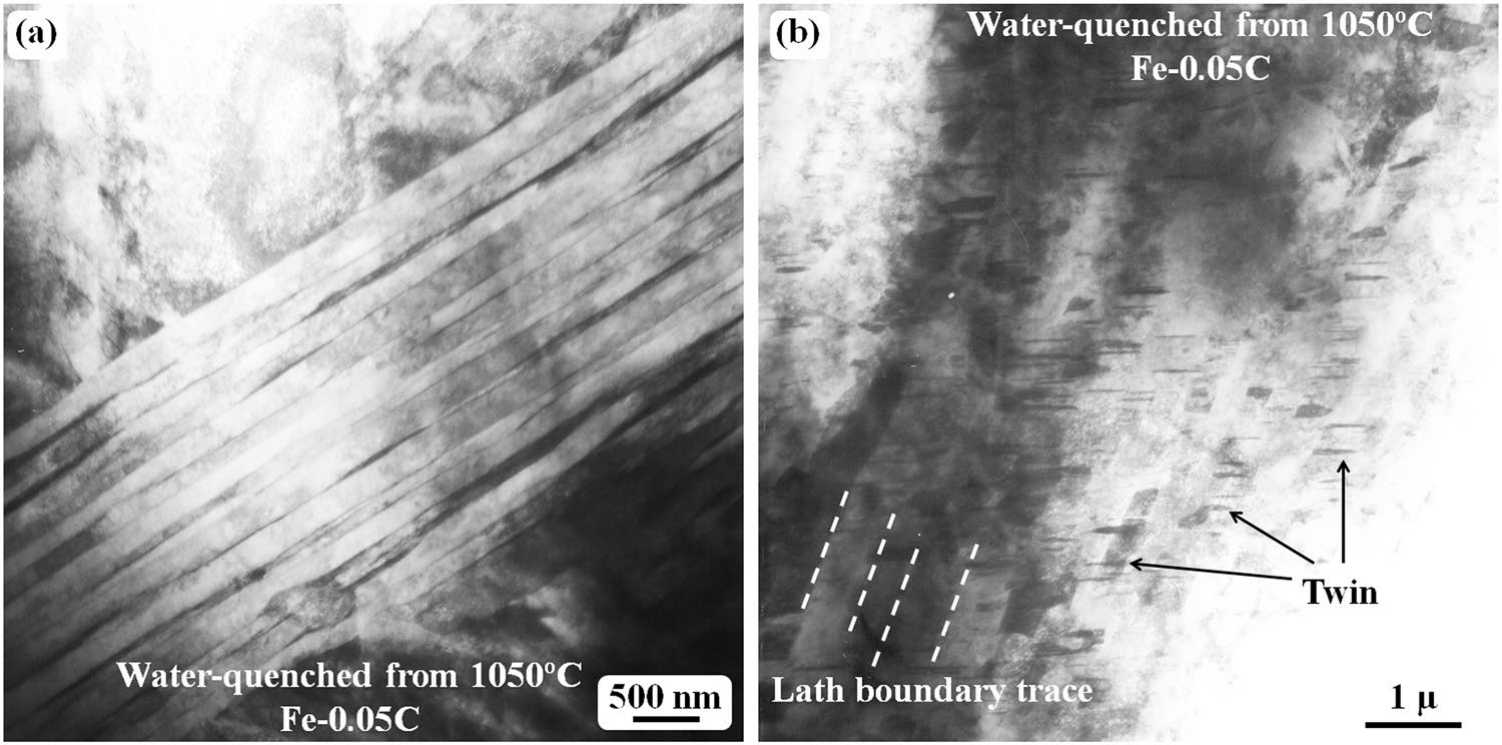
TEM bright-field images obtained from the Fe-0.05 C sample water-quenched at 1050 °C. (**a**) Lath martensite structure. (**b**) Short twins inside each lath. The trace of lath boundaries is indicated by white dashed lines.

When the austenitizing temperature was set to the range from 1050 °C to 1200 °C, the microstructure of the water-quenched samples was similar. Figure 8 shows the lath morphology of the quenched sample at 1050 °C. The general morphology (Fig. 8(a)) of the lath structure appears free of any carbides or twins. However, short twins inside the laths are also commonly observed as shown in Fig. 8(b). No twin contrast can be observed in Fig. 8(a), but this does not imply that no twins exist in these laths, as the twin contrast can only be observed from some special directions (the twinning plane is parallel to the incident electron beam).

## Discussion

All the TEM bright-field images reveal a typical lath microstructure in the water-quenched Fe-0.05 C samples. However, twins as the lath substructure or twins remaining inside laths were commonly observed at a temperature interval of 1050 °C~1200 °C. Interestingly, this temperature interval is popularly employed in the heat treatment of carbon steels in the engineering field.

As explained, decarbonization may occur, and thus, the samples subjected to higher-temperature treatment may contain less carbon. Although the laths observed in the samples quenched at higher temperature (1300 °C) are similar to those observed in samples quenched at lower temperature (950 °C)^11^, a significant difference has been observed in that there is a high density of fine cementite particles at the lath boundaries in the samples quenched at lower temperature (950 °C), whereas carbides are absent in the samples quenched at higher temperature in the present study, which suggests that decarbonization may occur during the higher-temperature treatment. The effects of austenitizing temperature on the water-quenched microstructures of the Fe-0.05 C samples are summarized in Table 1.

**Table 1:** Summary of the austenitized temperature effects on the water-quenched microstructures of the Fe-0.05 C samples.

Austenitized temperature	≤1050 °C (ref.^11^)	1050~1200 °C	≥1250 °C
Lath substructure	Dislocation-like	Twin + Dislocation-like	Dislocation-like
Lath boundary	Cementite particles	Twinned laths (with ω-Fe(C))	Fewer twinned laths
Mechanism	Auto-tempering effect	Retained initial martensite (twin structure) + auto-tempering effect	Decarbonization + auto-tempering effect

^*^All twinned boundary regions contain fine ω-Fe(C) particles.

As explained in our previous paper^11^, the twin relationship disappeared in the samples quenched at lower temperature (950 °C). The lost twinning relationship or structure is because auto-tempering has a pronounced effect on the detwinning process. Compared with the cooling rate of the sample subjected to a higher austenitizing temperature such as ~1200 °C, the sample quenched at 950 °C had a slower cooling rate when other quenching conditions were the same. Thus, the auto-tempering process occurred in the sample quenched at 950 °C for a longer time, and the detwinning could be completed.

**Figure 9:**
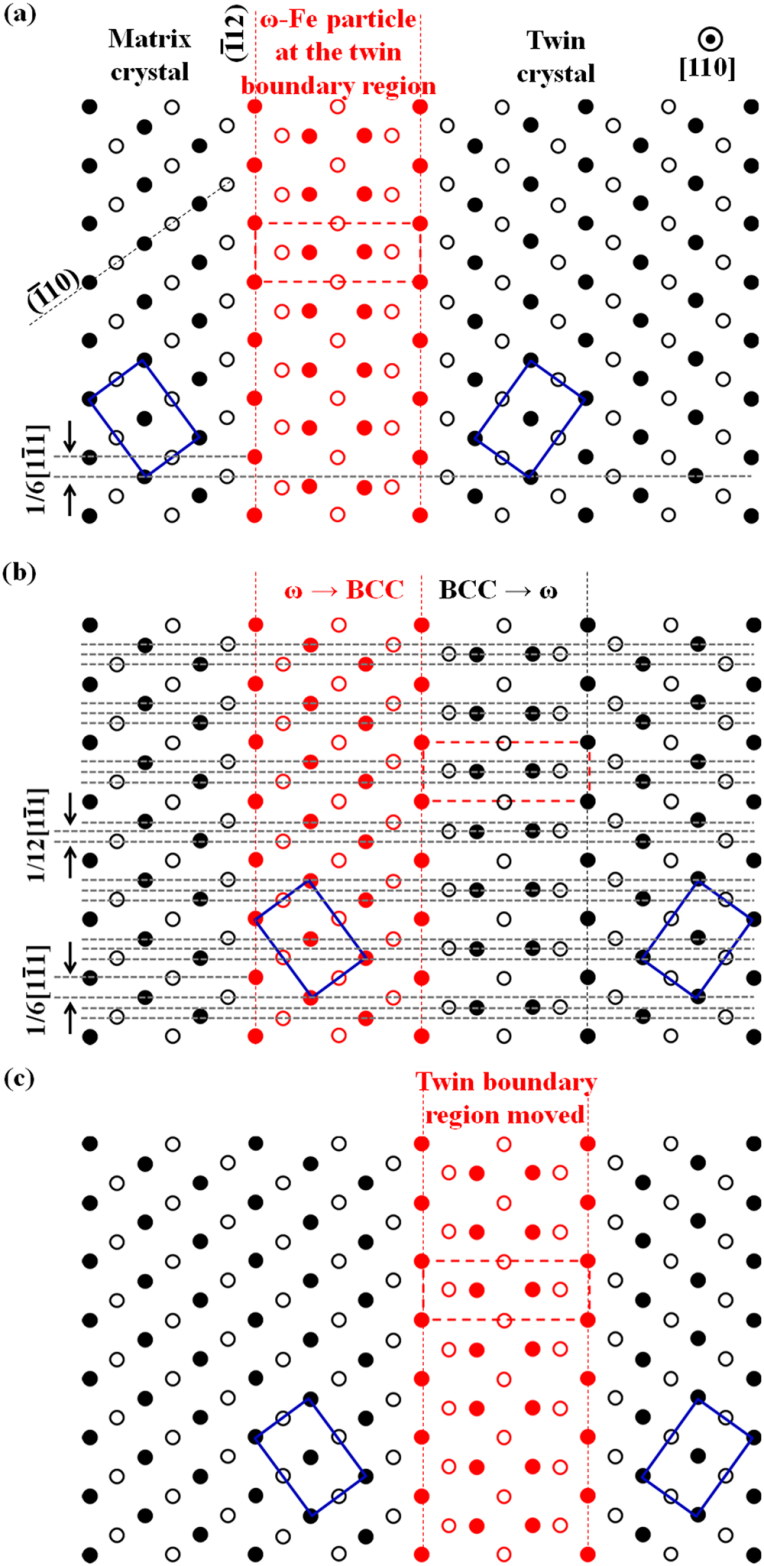
Schematic illustrations revealing the movement of the twinning boundary region. (**a**) The atomic model of a {$\bar{1}$12}〈1$\bar{1}$1〉 twin projected along the [110] direction. The twin boundary region contains the metastable ω-phase or fine ω-Fe particles. (**b**) The original ω-Fe at the original twin boundary region has transformed into the α-Fe (BCC) crystal; the surrounding BCC structure has transformed into the ω structure. (**c**) The transformation between the ω-Fe particles (ω-phase) and the α-Fe structure has resulted in the movement of the twin boundary region. The black dots and open circles represent the atoms at different layers. The twinning boundary movement is equal to the movement of the ω-Fe particles.

Theoretical calculation suggests that the energy gap between the ω and BCC structures is small; thus, the ω ↔ BCC transformation is not difficult^26^. As the {112}〈111〉-type twinning boundary always occurs with ultra-fine ω-Fe(C) particles in the present samples, the transformation between ω-Fe(C) and α-Fe may result in twinning boundary movement. The movement mechanism is illustrated in Fig. 9.

Figure 9(a) shows an atomic structure model of a {$\bar{1}$12}〈1$\bar{1}$1〉 twin in a BCC system. The model is projected along the BCC [110] direction, which is indicated in the right upper corner. The twin boundary region is the corresponding ω-structure. The unit cell of the ω-structure is outlined by red dashed lines. The unit cells of the BCC matrix and twin crystals are outlined by blue lines in the corresponding crystals.

Owing to the instability of ω-Fe(C) particles^23,26,28–32^, the ω ↔ BCC (ω-Fe ↔ α-Fe) transformation may occur at any time. One of the possible transformation results is shown in Fig. 9(b). The original ω phase (the original twin boundary region) in Fig. 9(a) has transformed into a BCC phase, and the right side of the original twin boundary region has transformed into a ω phase. Such types of transformation require a 1/12[1$\bar{1}$1] of atomic shuffling along the [1$\bar{1}$1] direction on the ($\bar{1}$12) plane.

Compared with the twin boundary region (Fig. 9(a)), the ω ↔ BCC (ω-Fe ↔ α-Fe) transformation has resulted in twin boundary movement, as shown in Fig. 9(c). Thus, the driving force for twin boundary migration is the instability of the ω-Fe phase. As ω-Fe particles are located on each twinning plane, all the twin boundaries move during the auto-tempering or post-tempering processes. Such twin boundary movement is also called the “detwinning” process, which can be treated as the opposite process to twin growth. Twin growth in BCC systems has been previously explained in detail^33^. Carbon atoms are assumed at the interstitial sites of the omega-phase (the twin boundary region), and this twin boundary movement can be equal to the carbon atom diffusion in martensite structure. Finally, the carbon atoms will segregate at the lath boundary region for the carbide formation. The evidence for this assumption can be found elsewhere^11,23,30,31^. This kind of carbon atom segregation may explain why carbides are preferably formed at the twinning boundary region^34^.

**Figure 10:**
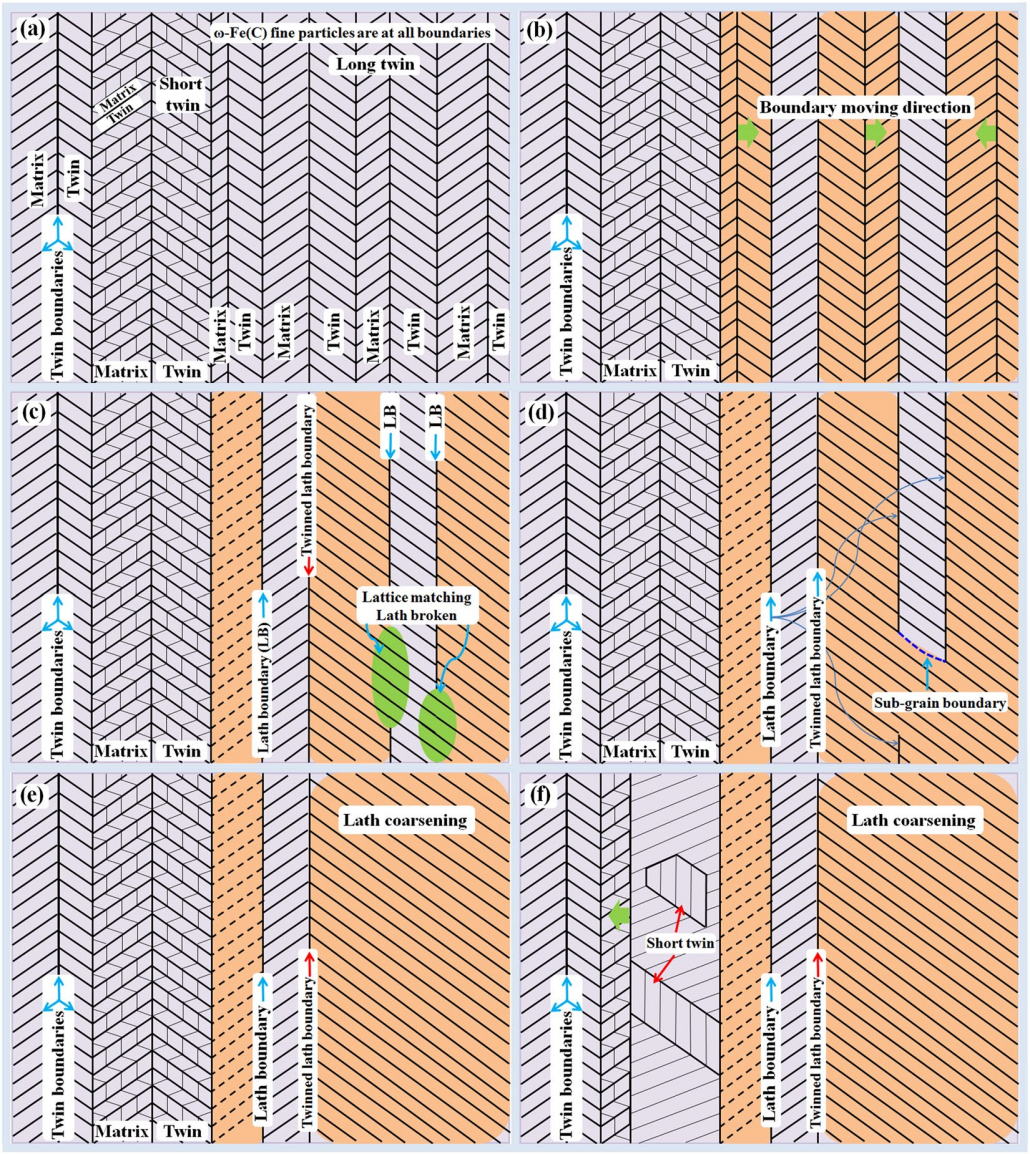
Two-dimensional schematic illustrations revealing the mechanisms of formation of laths, twinned laths, and short twins inside the laths in the water-quenched Fe-C alloys. (**a**) The {112} 〈111〉-type twinning structure as initially formed martensite structure. (**b**) Lath boundary moving directions. (**c**–**d**) Lath coarsening process. Lath may be broken during coarsening, thus becoming short. (**e**) No lath broken. Lath becomes thick. (**f**) Short twins remained inside the laths. The driving force for the boundary (all boundaries are twinned) movement is the instability of ω-Fe(C) fine particles at all twin boundaries or twinned lath boundaries.

With knowledge of the above twin boundary movement, it is much easier to understand the lath formation mechanism from the initial twinning structure in quenched martensite. Figure 10 shows the formation mechanisms of lath martensite, twinned laths and short twins inside laths. Notably, all twin boundaries contain unstable fine ω-Fe(C) particles. The coarsening process of the fine particles has been explained in a previous publication^11^. Here, the fine ω-Fe(C) particles are not shown for the sake of clarity in Fig. 10.

Figure 10(a) shows the initial martensite with a fully twinned structure. A twin structure with a metastable ω phase at the twinning boundary region as a product of martensitic transformation has been explained earlier^11,32^. Here, the twin, with its twinning boundary plane parallel to the lath boundaries, is designated the “long twin,” whereas the twins inside the laths are called the “short twins”. Figure 10(b) illustrates the possible twinning boundary directions of movement. Both left and right directions are possible. Following the movement directions, the lath crystals merge and become larger (Fig. 10(c)). During the boundary movement, carbides may form inside the laths, as explained in the literature^11^. The long laths may be broken at the region where the lattices match well, as shown in the green-colored region in Fig. 10(c,d). Simultaneously, twinned laths may also be formed. Figure 10(e) shows a thick lath formed during the twinning boundary movement. All these movements occurred during the auto-tempering process. Simultaneously, a detwinning process of the short twins occurs inside the laths (Fig. 10(f)). The long twin boundaries will finally become lath boundaries; however, the lath boundaries will not be affected by the detwinning of the short twins. The complete detwinning of these short twins will result in fine carbides entangled in dislocations or with sub-grain boundaries inside laths^35^. All twinning boundaries contain ω-Fe(C) particles; carbides may be formed when the twinning relationship is lost, as the stability of the ω-Fe(C) phase also relies on the twinning boundary structure^31^.

In summary, an Fe-0.05 C (wt.%) alloy was water-quenched at various austenitizing temperatures for 30 min. The quenched microstructures have been examined using TEM.

1. The lath martensitic substructure can be in the form of BCC {112}〈111〉-type twins in water-quenched Fe-0.05 C (wt.%) alloys at a temperature interval of 1050~1200 °C.
2. Above this temperature range, decarbonization may occur; the quenched microstructures thus observed mainly exhibit laths with a few or no twins.
3. Below this temperature range, carbides are formed at the lath boundaries via auto-tempering.

The formation mechanisms of laths, twinned laths, and short (internal) twins inside the laths have been explained based on the initial twinned martensite structure, and the driving force for this detwinning process during auto-tempering is the existence of an unstable ω-Fe(C) phase at the twinning boundary region.

The twin boundary movement discussed herein cannot be applied to the twin boundaries free of ω-phase; nevertheless, the BCC {112}〈111〉-type twin boundary is commonly occupied by the ω-phase. As the {112}〈111〉-type twin is very popular in BCC metals and alloys, the understanding of the twinning boundary structure and the detwinning mechanism is helpful for improving the mechanical properties of BCC metals and alloys, particularly carbon steels.

## Methods

A binary Fe- 0.05 C (wt.%) ingot was prepared in a high-vacuum induction furnace in an Ar atmosphere, and the ingot was solution-treated at approximately 1200 °C for 2 h and thereafter hot forged into a thick plate with a thickness of approximately 20 mm. Thin plates (~10 mm × 10 mm × 0.5~1.0 mm) were mechanically cut and austenitized at 1050~1350 °C for 30 min in a flowing Ar atmosphere, and thereafter quenched in water. The plates were directly placed in the furnace when the temperature reached the above mentioned value. The specimens for TEM observation were prepared from the water-quenched thin plates via mechanical grinding and polishing and subsequently ion-milled at room temperature. An ion mill (Fischione Model 1050 TEM Mill) was used for preparing the specimen at 4 kV. Microstructural observation was carried out using a JEM 2000FX TEM operated at 200 kV.
